# Use of Pyrazole Hydrogen Bonding in Tripodal Complexes to Form Self Assembled Homochiral Dimers

**DOI:** 10.3390/ma13071595

**Published:** 2020-03-31

**Authors:** Greg Brewer, Raymond J. Butcher, Peter Zavalij

**Affiliations:** 1 Department of Chemistry, Catholic University, Washington, DC 20064, USA; 2 Department of Chemistry, Howard University, Washington, DC 20059, USA; rbutcher@howard.edu; 3 Department of Chemistry and Biochemistry, University of Maryland, College Park, MD 20742, USA; pzavaliv@umd.edu

**Keywords:** supramolecular, dimer, pyrazole, hydrogen bonding, crystal structure, cobalt

## Abstract

The 3:1 condensation of 5-methyl-1H-pyrazole-3-carboxaldehyde (MepyrzH) with tris(2-aminoethyl)amine (tren) gives the tripodal ligand tren(MePyrzH)_3_. Aerial oxidation of a solution of cobalt(II) with this ligand in the presence of base results in the isolation of the insoluble Co(tren)(MePyrz)_3._ This complex reacts with acids, HCl/NaClO_4_, NH_4_ClO_4_, NH_4_BF_4_, and NH_4_I to give the crystalline compounds Co(tren)(MePyrzH)_3_(ClO_4_)_3_, {[Co(tren)(MePyrzH_0.5_)_3_](ClO_4_)_1.5_}_2_ {[Co(tren)(MePyrzH_0.5_)_3_](BF_4_)_1.5_}_2_ and [Co(tren)(MePyrzH)_3_][Co(tren)(MePyrzH)_3_]I_2._ The latter three complexes are dimeric, held together by three N_pyrazole_ –H^…^N_pyrazolate_ hydrogen bonds. The structures and symmetries of these homochiral dimers or pseudodimers are discussed in terms of their space group. Possible applications of these complexes by incorporation into new materials are mentioned.

## 1. Introduction

**Figure 1:**
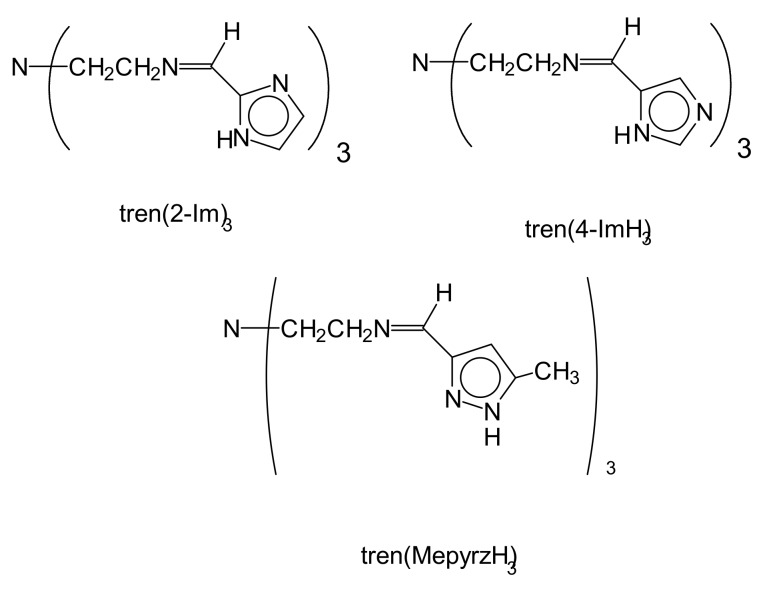
Line drawings of tripodal azole ligands.

Tripodal ligands (see Figure 1) discussed in this report are formed from the Schiff base condensation of tris(2-aminoethyl)amine (tren), with three moles of an imidazole-2-carboxaldehyde, imidazole-4-carboxaldehyde or 5-methyl-1H-pyrazole-3-carboxaldehyde. The imidazole rings may have methyl substituents (not illustrated) in the 1,2 or 4 positions, which does not drastically affect reactivity [1,2]. The ligands are triprotic, H_3_L, and can coordinate to a metal(II) or metal(III) to give mononuclear complexes [MH_3_L]^2+^, [MH_3_L]^3+^ or [ML] in the presence of base [3,4,5,6,7]. The resultant imidazole and imidazolate complexes have been extensively studied in terms of proton coupled electron transfer (PCET) [8,9,10,11,12,13], tunable redox potentials, spin crossover (SC) between spin(HS) low spin(LS) electronic ground states [14,15,16] and formation of double salts which exhibit size recognition properties [17,18]. The properties of the metal complexes of the fully protonated (H_3_L) or fully deprotonated ligands (L^3−^) are certainly important as they have far ranging applications to materials research including switches [19,20], memory storage [21], and magnets [22,23]. The complexes are also chiral as the three arms of the tren ligand can wrap around the metal in a clockwise or counter clockwise fashion to give Δ (delta) or Λ (lambda) complexes.

The supramolecular [24,25,26] properties of the complexes of partially deprotonated or hemideprprotonated ligands (H_2_L^−^, HL^2−^, H_1.5_L^−1.5^) are perhaps more important in potential materials science applications. These complexes self assemble to give extended molecular arrays that exhibit very different topologies and preference for homo vs heterochirality.

**Figure 2:**
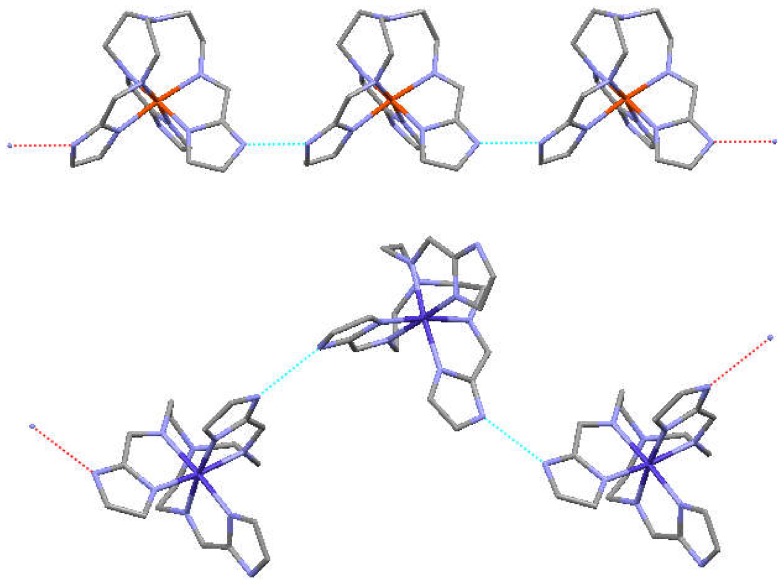
Partially deprotonated complexes have been observed for [Fetren(2-Im)_2_(2-ImH)]^+^ (linear, top) and [Cotren(2-ImH)_2_(2-Im)]^2+^ (zig-zag, bottom) that give 1D hydrogen bound chains. Please see [27] from preceding paragraph.

The driving force is the formation of an extensive network of hydrogen bonding interactions between a protonated azole and a deprotonated azole, N_azole_–H^…^N_azolate_. Examples of complexes of H_2_L^−^ and HL^2−^ ligands that give 1D linear and zig-zag chains are illustrated below in Figure 2 [27]. 

**Figure 3:**
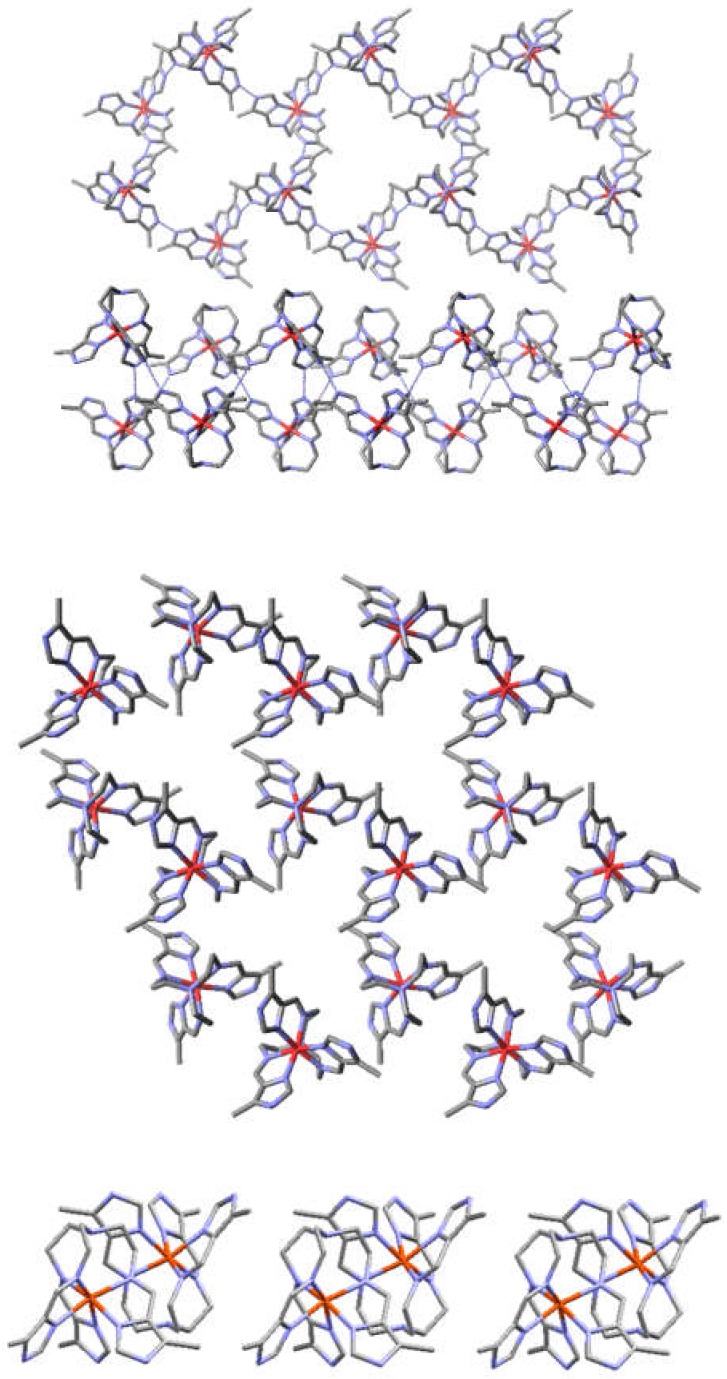
Face on upper and sideway lower views of extended 2D sheet structures for trigonal Fetren(4-MeImH_0.5_)_3_^+1.5^ (top) and hexagonal [Fetren(4-MeImH_0.5_)_3_]^+1.5^ (bottom). Please see [31] from preceding paragraph.

The greatest number of supramolecular complexes are those of the hemideprotonated ligand with with iron(II), iron(III), cobalt(II) or cobalt(III) as the metal. The hemideprotonated state can be achieved if each of the three N1 azole nitrogen atoms has a hydrogen atom at half occupancy, NH_0.5_, tren(azoleH_0.5_)_3_^−1.5^ or if the metal complexes average out to 1.5 hydrogen atoms per complex as in a 50:50 compound of [Mtren(azoleH)_3_]^+2 or +3^ and [Mtren(azole)_3_]. The hemideprotonated tren(2-ImH_0.5_)_3_^−1.5^ and tren(4-ImH_0.5_)_3_^−1.5^ systems exhibit a 2D hexagonal sheet structure, which exhibit extensive hydrogen bonding between neighboring complexes [28]. Each hemideprotonated complex is hydrogen bound to three neighboring structures, which give an extended 2D sheet. There is little or no interaction between adjacent layers [29,30]. Examples of these are depicted in Figure 3 [31].

This work describes the preparation and structures of the cobalt complexes of the tren(MepyrzH)_3_ ligand.

## 2. Experimental

### 2.1. General Information

Tris(2-aminoethyl)amine (tren), cobalt(II) tetrafluoroborate hexahydrate, cobalt(II) perchlorate hexahydrate, ammonium perchlorate, ammonium tetrafluoroborate, ammonium iodide and 0.10 M potassium hydroxide in methanol were obtained from Aldrich (Milwaukee, WI, USA). 5-Methyl-1H-pyrazole-3-carboxaldehyde was obtained from ChemBridge (San Diego, CA, USA). All solvents were of reagent grade and used without further purification. IR spectra were obtained as KBr pellets on a 1600 FT IR spectrometer (Perkin Elmer, Walthem, MA, USA).

### 2.2. Synthesis

[Co(tren(MepyrzH)_3_](ClO_4_)_3_ A solution of HCl (1.32 mL of 0.1 M HCl in methanol, 0.132 mmol) was added to a slurry of the previously prepared [32] Cotren(Mepyrz)_3_ (0.021 g, 0.044 mmols) in methanol (40 mL) in a 100 mL round bottom flask. The mixture was refluxed for an hour and filtered while hot. An excess of NaClO_4_ in a few mL of methanol was added. Orange-red crystals precipitated overnight.

{[Cotren(MepyrzH_0.5_)_3_](ClO_4_)_1.5_}_2_ NH_4_ClO_4_ (0.034 g, 0.29 mmol) was added as a solid to a slurry of Cotren(Mepyrz)_3_ (0.023 g, 048 mmol) in methanol (30 mL) in a 100 mL round bottom flask. There was no immediate change. The mixture was refluxed for 2 h to give a clear, orange solution. The reaction mixture was filtered while hot and set aside to concentrate. After several hours small red crystals were produced. They were recrystallized from DMF to produce crystals suitable for crystallography.

{[Cotren(MepyrzH_0.5_)_3_](ClO_4_)_1.5_}_2_ NH_4_BF_4_ (0.029 g, 0.28 mmol) was added as a solid to a slurry of Cotren(Mepyrz)_3_ (0.021 g, 044 mmol) and methanol (30 mL) in a 100 mL round bottom flask. There was no immediate change. The mixture was refluxed for 2 h to give a clear, orange solution. The reaction mixture was filtered while hot and set aside to concentrate. After several hours small red crystals were produced. Two different types of crystals, the title compound and a hydrate) were produced and analyzed 

[Cotren(MepyrzH)_3_][Cotren(Mepyrz)_3_]I_2_ NH_4_I (0.047 g, 0.32 mmol) was added as a solid to a slurry of Cotren(Mepyrz)_3_ (0.032 g, 067 mmol) and methanol (10 mL) in a 100 mL round bottom flask. There was no immediate change. The mixture was refluxed for 2 h to give a clear, orange solution. The reaction mixture was filtered while hot and set aside to concentrate. After several hours small red crystals were produced.

{[Cotren(MepyrzH_0.5_)_3_ (C_6_H_6_) CH_3_CN)}_2_(ClO_4_)_3_. This complex was isolated in an attempt to repeat the synthesis of [Cotren(MepyrzH)(Mepyrz)_2_](ClO_4_) [33]. Tren (0.077 g, 0.53 mmol) and absolute ethanol (~16 mL) were added to a 25 mL round bottom flask. 5-Methyl-1H-pyrazole-3-carbaldehyde (0.174 g, 1.6 mmol) was added as a solid. The reaction mixture was refluxed for 2 h and filtered. Co(ClO_4_)_2_^.^6H_2_O (0.193, 0.53 mmol) was added as a solid to the filtrate. The reaction mixture was refluxed a further 2 h and was set aside to concentrate. Over a week a large quantity of solid had formed. It was filtered, washed sparingly with absolute ethanol and placed in a dessicator over CaCl_2_ to dry. After several days the solid (0.110 g) was dissolved in 15–20 mL acetonitrile with warming. The solution was filtered into a beaker. The beaker was set in a sealed jar with a few mL benzene in jar to allow for slow diffusion of benzene into the acetonitrile solution. Large brown blockish crystals formed over several days.

### 2.3. Structure Determinations

Crystal data were collected on a SMART 1000 CCD area2 detector Apex II system (Bruker, Madison, WI, USA), or on an Oxford Gemini diffractometer (Oxford, UK). All structures were solved using the direct methods program SHELXS-97 (Univ. of Gottingen, Germany) [34]. All nonsolvent heavy atoms were located using subsequent difference Fourier syntheses. The structures were refined against F^2^ with the program SHELXL [35,36], in which all data collected were used including negative intensities. In several of the complexes the perchlorate or tetrafluoroborate anions were conformationally disordered. In these cases each conformation was tetrahederally idealized and the multiplicities of the conformations were constrained to unity. All nonsolvent heavy atoms were refined anisotropically. All hydrogen atoms were located by Fourier difference. Complete crystallographic details are summarized in Table 1. Selected bond distances and angles are given in Table 2 and hydrogen bonding data is in Table 3. Crystallographic data for all complexes can be obtained free of charge from the Cambridge Crystallographic Data Center. The deposition numbers for the complexes are provided. [Cotren(MepyrzH)_3_] (ClO_4_)_3_ 973179, [Cotren(MepyrzH_0.5_)_3_]_2_ (ClO_4_)_3_ 973180, [Cotren(MepyrzH_0.5_)_3_]_2_ (BF_4_)_3_ 973182, [Cotren(MepyrzH)_2_(Mepyrz)] [Cotren(Mepyrz)_2_(MepyrzH)] (BF_4_)_3_^.^3.81 H_2_O 973183, {[Cotren(MepyrzH_0.5_)_3_ (C_6_H_6_) CH_3_CN)}_2_(ClO_4_)_3_ 973181 (123 K), {[Cotren(MepyrzH_0.5_)_3_ (C_6_H_6_) CH_3_CN)}_2_(ClO_4_)_3_ 973178 (150 K), [Cotren(MePyrzH)_3_][Cotren(MePyrz)_3_]I_2_ 973177.

## 3. Results and Discussion

### 3.1. Synthesis and Initial Characterization

The syntheses of [Cotren(MePyrzH)_3_](ClO_4_)_3_, [Cotren(MePyrzH_0.5_)_3_]_2_(ClO_4_)_3_ and [Cotren(MePyrzH_0.5_)_3_]_2_(BF_4_)_3_ were achieved by the reaction of the previously prepared insoluble Co(tren)(MePyrz)_3_, [37] with Lewis acids as illustrated below. The insolubility of this species is not understood in comparison to analogous complexes of similar ligands, but does not appear to hinder its reactivity. Lewis acid base reactions can be conducted by adding a solution of a suitable Lewis acid to a slurry of [Cotren(MePyrz)_3_] which acts as a Lewis base due to its three deprotonated pyrazolate rings. The resulting solution of both reactants isleft to stand and the products crystallize from the reaction mixture.

Cotren(MePyrz)_3_(s) + 3 HCl/NaClO_4_ (aq/methanol) → [Cotren(MePyrzH)_3_](ClO_4_)_3_

2Cotren(MePyrz)_3_(s) + 3NH_4_ClO_4_ (methanol) → [Cotren(MePyrzH_0.5_)_3_]_2_(ClO_4_)_3_ + 3NH_3_ (1)

2Cotren(MePyrz)_3_(s) + 3NH_4_BF_4_ (methanol) → [Cotren(MePyrzH_0.5_)_3_]_2_(BF_4_)_3_ + 3NH_3_

The tetrafluoroborate salt crystallizes as both an anhydrous and a hydrated form and both were structurally characterized. The synthesis of the analogous iodide salt is complicated by the fact that the iodide ion can reduce the cobalt(III) to cobalt(II). The product is a cobalt(II)-cobalt(III) mixed valence species [38], [Cotren(MePyrzH)_3_][Cotren(MePyrz)_3_]I_2_. The likely sequence of reactions is protonation of the cobalt complex by the ammonium cation, followed by reduction of this species by iodide and the formation of the pseudo dimer as illustrated below:

2Cotren(MePyrz)(s) + 6NH_4_I → 2[Cotren(MePyrzH)_3_](I)_3_ + 6NH_3_

2[Cotren(MePyrzH)_3_](I)_3_ → 2[Cotren(MePyrzH)_3_](I)_2_ + I_2_

NH_4_I + I_2_ → NH_4_I_3_ (2)

2[Cotren(MePyrzH)_3_](I)_2_ + 2[Cotren(MePyrz)_3_] → 2[Cotren(MePyrzH)_3_][Cotren(MePyrz)_3_](I)_2_

4Cotren(MePyrz)(s) + 7NH_4_I → 2[Cotren(MePyrzH)_3_][Cotren(MePyrz)_3_]I_2_ + 6NH_3_ + NH_4_I_3_

For completeness an attempt was made to prepare the previously synthesized [33] and structurally characterized [Cotren(MepyrzH)(Mepyrz)_2_](ClO_4_) by following the literature preparation which grew crystals by infusion of benzene into an acetonitrile solution of the reaction product. The present work resulted in the isolation of {[Cotren(MepyrzH_0.5_)_3_ (C_6_H_6_) CH_3_CN)}_2_(ClO_4_)_3_ despite careful attempts to reproduce the previously reported synthesis. The present inability to isolate the reported [Cotren(MepyrzH)(Mepyrz)_2_](ClO_4_) is not understood, but it is possible that in the earlier work the purification produced both [Cotren(MepyrzH)(Mepyrz)_2_](ClO_4_) (which was reported) and {[Cotren(MepyrzH_0.5_)_3_ (C_6_H_6_) CH_3_CN)}_2_(ClO_4_)_3_ which was not reported or observed earlier. However, it is isolated and structurally characterized as part of this work.

The initial characterization of complexes was carried out by IR spectroscopy. The most useful regions for examination in the IR were the N-H, C=N, and perchlorate Cl-O, and tetrafluoroborate B-F. The IR spectra of the [Cotren(MepyrzH)_3_](ClO_4_)_3_ mononuclear complex shows the characteristic absorption bands attributable to the pyrazole ν_N-H_ (3100–3300 cm^−1^), imine ν_C=N_ at ~1640 cm^−1^ and perchlorate ν_Cl-O_ (1147–1154 and 625 cm^−1^). The position of the imine absorption for the cobalt complexes increases with the protonation state of the ligand [39]. For [Cotren(Mepyrz)_3_] the absorption appears close to 1600 cm^−1^, consistent with deprotonation of the ligand. This peak shifts to higher wavenumbers for the dimers/pseudodimers. The [Cotren(MePyrzH)_3_][Cotren(MePyrz)_3_]I_2_ pseudo-dimer exhibit two imine ν_C=N_ absorptions in the IR spectrum corresponding to the Co(II) and Co(III) components. In addition to the expected ν_N-H_ and ν_C=N_ absorptions, the IR spectra of all the pseudo-dimers exhibit broad bands at ~2100–1800 and ~2375 cm^−1^, which are not observed in the IR spectra of the mononuclear complexes. These bands have been observed previously in similar hydrogen bound complexes and are attributed to intermolecular azole-azole hydrogen bonding [40,41,42].

### 3.2. Structures of the Complexes, General Features

**Figure 4:**
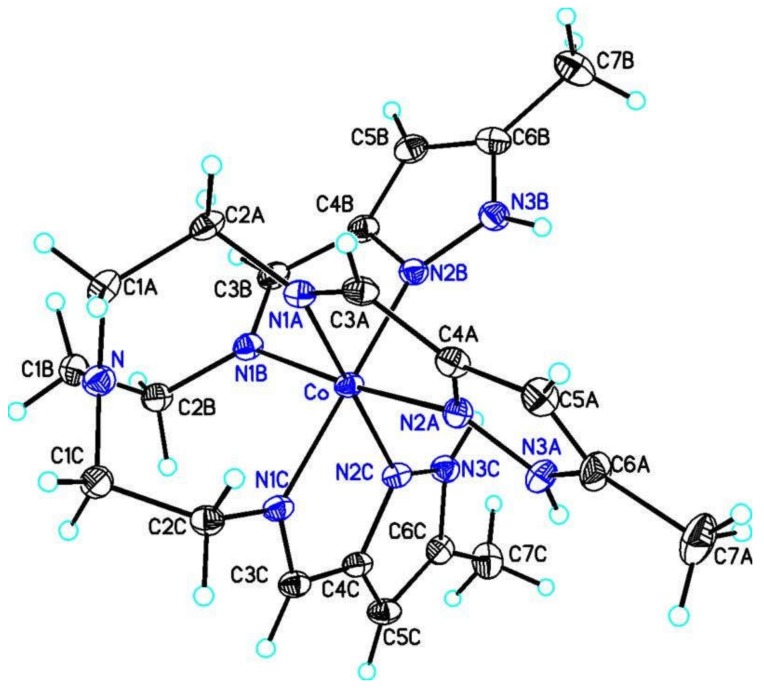
Structure of the [Co(tren(MepyrzH)_3_]^3+^ cation. Note that the apical nitrogen atom, labelled N above, caps the face of the three imine nitrogen atoms, N1A, N1B and N1C.

All of the cobalt species reported here are six coordinate distorted octahedral complexes bound to three facial pyrazole nitrogen atoms and three facial imine nitrogen atoms. The apical nitrogen atom (N_ap_) of the tren caps the three facial imine nitrogen atoms. In these complexes the Co to N_ap_ distance is too large to be considered a bond. However in related complexes the distance is short enough that the geometry is the seven coordinate capped octahedron [43]. The complexes are chiral, Λ or Δ, as determined by the twist orientation of the three tren arms. Relevant bond distance and angles are given for all complexes in Table 2. The above features are illustrated for [Cotren(MepyrzH)_3_](ClO_4_)_3_ in Figure 4. This is the only mononuclear complex reported here and the remaining complexes are dimeric or pseudodimeric species held together by three N_pyrazole_–H^…^N_pyrazolate_ hydrogen bonds as described below. There are earlier reports of mononuclear tren pyrazole complexes [44,45,46,47].

### 3.3. General Structure of the Dimers/Pseudodimers

**Figure 5:**
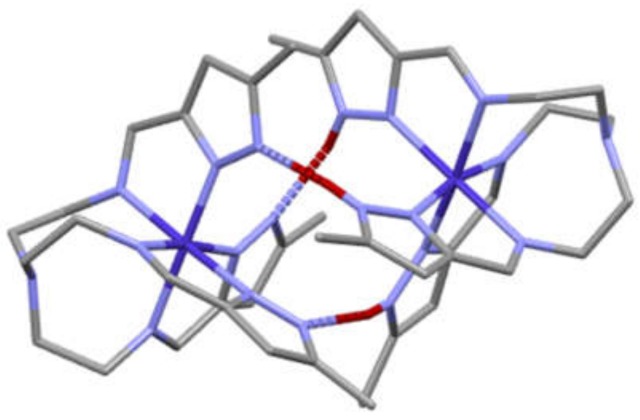
Structure of the [Cotren(MepyrzH)_3_][Cotren(Mepyrz)_3_]^2+^ cation. All the hydrogen atoms except for the bridging hydrogen atoms, shown in red, have been deleted. Note the three.N_pyrazole_–H^…^N_pyrazolate_ hydrogen bonds, shown as dashed blue red bonds, holding the two homochiral halves of cation together and the alignment of the three pairs of pyrazole rings to promote π-π stacking. The apical nitrogen atoms of the tren units are in light blue at the bottom left and upper right of figure. The two octahedral cobalt atoms are in dark blue, the cobalt(III) on left and cobalt(II) on right.

Structures of several tren pyrazolate dimers or pseudodimers, [Fetren(MepyrzH)_3_–Fetren(Mepyrz)_3_]^2+^, [Mntren(MepyrzH)_3_–Fetren(Mepyrz)_3_]^2+^, [Fetren(MepyrzH)_3_–Cotren(Mepyrz)_3_]^2+^ [Mntren(MepyrzH)_3_–Cotren(Mepyrz)_3_]^2+^ have been reported previously [32,37]. The term dimer is used if there is a single metal complex in the asymmetric unit, meaning that it is a true dimer, made of two identical halves Even in a dimer that contains two different metals, M and M’, or the same metal in two different oxidation states the symmetry imposed by the space group may average these which manifests as a M_0.5_ M’_0.5_ bound to a hemideprotonated ligand, tren(MepyrzH_0.5_)_3_^−1.5^. The term pseudodimer is used if there are two metal complexes in the asymmetric unit. This too is determined by the symmetry imposed by the space group. In this case the two metal complexes are different, either because the metals are different, in different oxidation states or the levels of protonation on the two ligands are different, such as tren(MepyrzH)_3_. tren(Mepyrz)_3_^3−^. Regardless of this distinction between dimer and pseudodimer both complexes exhibit three structural features in common. 1) The dimers have three N_pyrazole_–H^…..^N_pyrazolate_ hydrogen bonds that link the two halves of the dimer/pseudodimer together. 2) The dimers exhibit π-π stacking of the three pairs of pyrazole rings. The pyrazole rinds are not perfectly eclipsed. There is slippage between the two rings but their small interplanar angle (<2°) and a short centroid to centroid distance (~3.4 Å) supports the π-π interaction. And 3) homochirality of both metal complexes of the dimer/pseudodimer, either both Δ or both Λ. These features are illustrated in Figure 5 for [Cotren(MepyrzH)_3_][Cotren(Mepyrz)_3_]^2+^ cation. This homochirality feature is required for the formation of both the hydrogen bonding and the π-π stacking. There are other examples of complexes that exhibit some of these features individually. A copper Schiff base dimer is held together by hydrogen bonding but lacks the other structural elements [48]. The π-π stacking feature is observed in imidazole based supramolecular complexes of manganese(II) and cobalt(II) [49]. The intervalence and hydrogen bonding features of the Co(II)-Co(III) pseudodimer were observed in a ferrocene derivative [50]. The structures of the present cobalt complexes exhibit these same features but these new systems exhibit previously unobserved symmetry characteristics due to the crystallization of the complex in Sohncke [51] space groups as discussed in the descriptions that follow.

**Figure 6:**
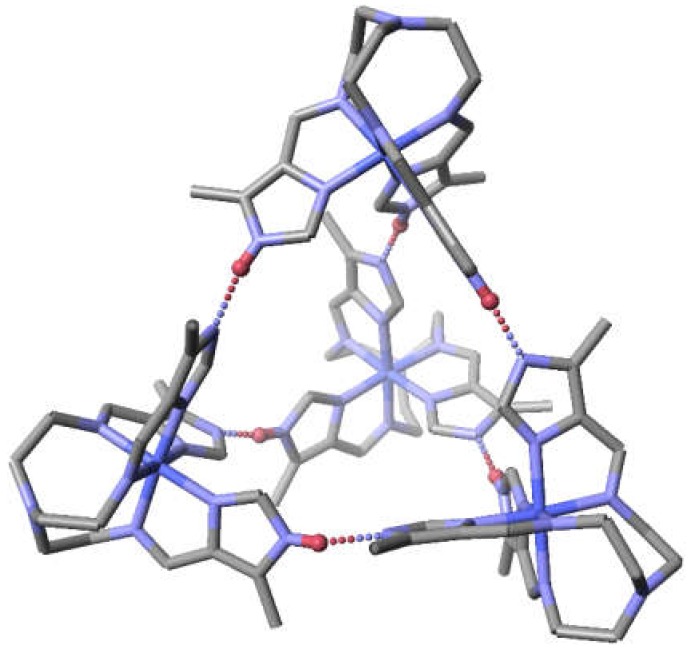
Structure of [Cotren(4MeImH_0.5_)_3_]_4_(ClO_4_)_6_ (average level of protonation per Co) showing the tetrahedral array of cobalt complexes and the six hydrogen bonds folding the cluster together. All hydrogen atoms, except for the hydrogen bound bridging hydrogen atoms, shown in red, and the perchlorate anions have been omitted for clarity. Note that there are twelve hydrogen bound imidazole atoms which require six hydrogen atoms to link all the imidazoles together. Each cobalt complex forms three hydrogen bonds (through its three arms) to each of the other three cobalt complexes.

In the case of the tren pyrazole dimers both halves of the dimer must be of the same chirality either delta or lambda in order for the hydrogen bonding and π-π stacking to occur. Another example of polynucleation that must select for homochirality is the tetrahedral tetranuclear complex, {[Cotren(4-MeImH)(4-MeIm)_2_]_3_[Cotren(4-MeImH)_3_]}(ClO_4_)_6,_ which is averaged as [Cotren(4-MeImH_0.5_)_3_]_4_(ClO_4_)_6,_ and pictured in Figure 6 [52]. In the case of both the dinuclear and tetranuclear complex homochirality within the polynuclear unit, dimer or tetramer, is required to form the three (dimer) or six hydrogen bonds (tetramer) that hold the polynuclear unit together. In both cases each monomer forms three hydrogen bonds. In the dimer the three hydrogen bonds are to the same molecule and in the tetramer the three hydrogen bonds are to three different complexes. Extension of homochirality throughout the entire crystal can only occur in a Sohncke space group which is non-centrosymmetric and contains a single enantiomer.

Broadly speaking there are two types of symmetry elements that could be observed in these Mtren(Mepyrz)_3_ dimers/pseudodimers. A three-fold axis along the apical tren nitrogen atom and the metal atom (making the three tren arms identical) or a symmetry element that interchanges the two metal sites such as a two fold rotation axis. The former would result in a single tren arm per metal complex in the asymmetric unit and the latter would give three tren arms but an average metal site, [M_0.5_ M_0.5_’L]. The presence of both symmetry elements would give a single tren arm and a single metal atom in the asymmetric unit. Some previously prepared dimers/pseudodimers crystallized in C_2/c_ (C_2h_) and P_bcn_ (D_2h_). These space groups are centrosymmetric, contain both enantiomers and exhibit one metal and three tren arms in the asymmetric unit due to a two fold proper rotation axis. Others crystallize in C_c_ (C_s_) (non centrosymmetric, both enantiomers) and Pbar1 (C_i_) (centrosymmetric, both enantiomers). The dimers in these groups exhibit two metal sites and three tren arms/metal in the asymmetric unit. None of these space groups are of the Sohncke classification. In the present collection of homonuclear cobalt complexes Sohncke space groups are observed for three of the dimers which alters the symmetry considerations significantly.

**Figure 7:**
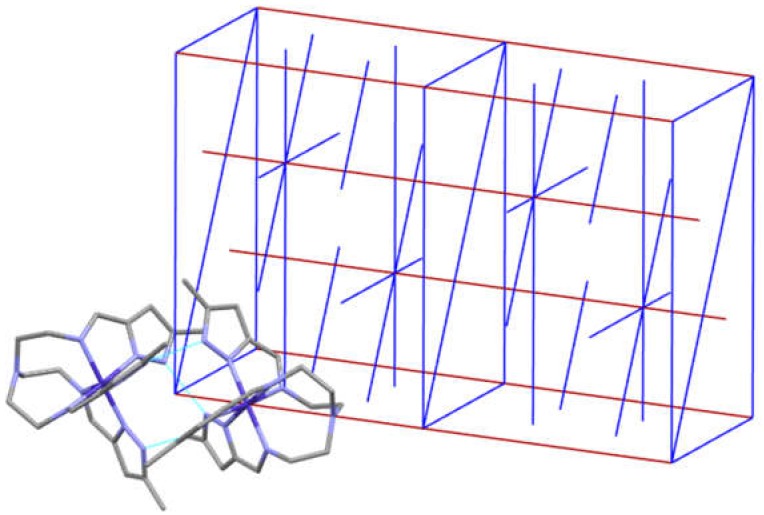
The unit cell (space group R32) of [Cotren(MepyrzH_0.5_)_3_]_2_^3+^ is depicted above with one of the dimers (all H atoms and counterions omitted for clarity) that it contains. The red line is a three fold rotation axis and also an axis of the cell. Note that the three fold axis (red) runs through the apical tren nitrogen and cobalt atoms which result in a single tren arm in the asymmetric unit. R32 (D_3_) requires a three fold rotation axis (red) and three perpendicular two fold rotation axis (blue). The two fold rotation (blue) bisects the non bonded cobalt-cobalt axis and is perpendicular to a N_pyrazole_–H^…^N_pyrazolate_ hydrogen bond. This results in a single cobalt atom in the asymmetric unit by a two fold rotation. The effect of both of these symmetry elements is that the asymmetric unit contains a single cobalt atom (not two) and a single tren arm (not three).

[Cotren(MepyrzH_0.5_)_3_]_2_ X_3_ (X = ClO_4_^−^ and BF_4_^−^). The structures of [Cotren(MepyrzH_0.5_)_3_]_2_ X_3_ (X = ClO_4_^−^ and BF_4_^−^) dimers are isomorphous in Sohncke space group R32 which has D_3_ symmetry. There is a single cobalt(III) ion and a three fold rotation axis (shown in red) that runs through the apical tren nitrogen and the cobalt atoms. They also possess three two fold rotation axis (shown in blue) that bisects the cobalt-cobalt non bonded axis. This is illustrated in Figure 7.

There are no mirror planes or glide planes as R32 is a Sohncke space group that allows for the crystallization of a single enantiomer of a chiral molecule. A mirror plane is equivalent to an improper axis of rotation which a chiral molecule cannot have. This type of symmetry, imposed by a Sohncke space group, has not been observed previously in the earlier pyrazole dimers/pseudodimers. The entire crystal is homochiral (resolved at the level of the crystal) but the entire sample is likely achiral as it contains equal numbers of molecules of opposite chirality (racemic conglomerate). Crystals of this type could be separated by hand into the lambda and delta isomers as was done by Pasteur for tartrate salts.

[Cotren(MepyrzH_0.5_)_3_]_2_ (BF_4_)_3_ also crystallizes as a hydrate, [Cotren(MepyrzH)_2_(Mepyrz)] [Cotren(Mepyrz)_2_(MepyrzH)] (BF_4_)_3_^.^3.81 H_2_O, in Cc. It is neither isomorphous or a polymorph of [Cotren(MepyrzH_0.5_)_3_]_2_. In this case the space group requires that the two metal sites be distinct and that there is no three fold axis along the apical tren nitrogen and cobalt atoms. These differences are achieved by having different levels of protonation on the two pyrazolate rings of the two ligands in the pseudodimer to give the {[Cotren(MepyrzH)_2_(Mepyrz)] [Cotren(MepyrzH)(Mepyrz)_2_]}^3+^ cation.

**Figure 8:**
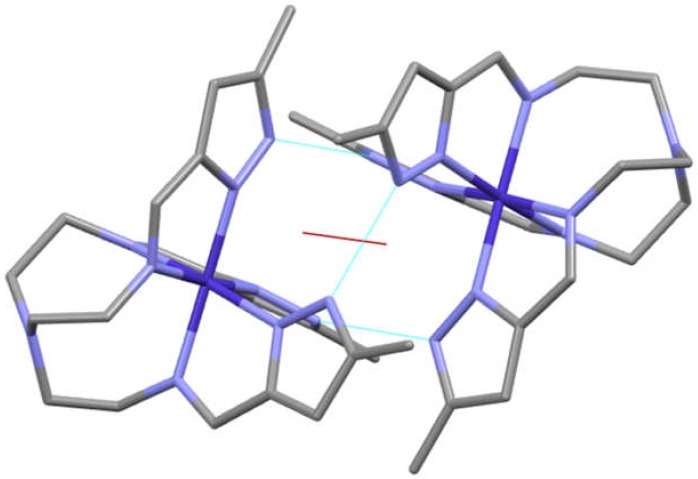
Structure of the pyrazole dimer in P4_3_2_1_2. The unit cell axes are not depicted here. The hydrogen atoms and counterions have been omitted for clarity. Note the three hydrogen bonds (dashed blue) connecting the tree pairs of pyrazole rimgs and the alignment of rings for π-π stacking. The red line is a C_2_ proper rotation axis that bisects one of the three hydrogen bonds and the non bonded cobalt-cobalt axis. This two fold rotation axis (the only symmetry element in the molecule) means that there is a single cobalt atom and three tren arms in the asymmetric unit. This contrasts with the previous molecule in R32 in that it lacks a three fold rotation axis on the molecule. The four fold rotation axis in this space group (D_4_ symmetry) is not a rotation axis of the molecule itself but a screw axis of the cell. Clerly a tripodal ligand could not have a fold fold proper rotation axis.

{[Cotren(MepyrzH_0.5_)_3_ (C_6_H_6_) CH_3_CN)}_2_(ClO_4_)_3_. This complex resulted from the attempt to repeat the earlier synthesis of [Cotren(MepyrzH)(Mepyrz)_2_]ClO_4_. This attempt resulted in the preparation of a new compound that crystallized in P4_3_2_1_2 which has D4 symmetry. In this dimer the two cobalt complexes are equivalent as a two-fold rotation axis bisects a N_pyrazole_–H^…..^N_pyrazolate_ hydrogen bond axis and the non-bonded cobalt-cobalt axis as shown in Figure 8. The three tren arms are not equivalent. The four fold rotation axis is a screw axis of the cell (not the molecule) There are no mirror planes as this is a Sohncke space group that allows for crystallization of a single enantiomer as was discussed earlier for [Cotren(MepyrzH_1.5_)_3_]_2_ X_3_ (X = ClO_4_^−^ and BF_4_^−^) that crystallizes in R32 (racemic conglomerate). The situation here is more complicated as P4_3_2_1_2 is an entaniomorphous (chiral) space group which means that if one enantiomer crystallizes in P4_3_2_1_2 the other enantiomer cannot crystallize in the same space group and must crystallize in the enantiomorphous space group, P4_1_2_1_2 in this case. This could result in total spontaneous resolution. While it was not possible to examine each crystal a structural determination was made of another crystal from the same batch and it was identical to the first. Both results are reported here.

[Cotren(MePyrzH)_3_][Cotren(MePyrz)_3_]I_2_. This compound was prepared by the same method as were [Cotren(MepyrzH_0.5_)_3_]_2_ X_3_ (X = ClO_4_^−^ and BF_4_^−^)_._ The reaction was simply adding a solution of ammonium iodide (rather than perchlorate or tetrafluoroborate) to the insoluble Cotren(Mepyrz)_3_. The result in this case was different as the product was the mixed valent cobalt(II)-cobalt(III) pseudodimer in Cc (Cs symmetry). There are two cobalt complexes in the asymmetric unit each with three unique tren arms. In this case one complex has all three pyrazolates protonated and the other has all three deprotonated. These are assigned as cobalt(II) and cobalt(III) respectively due to the fact that reduction is easier for a positively charged species over a neutral assuming other factors are unchanged. This species is an intervalence compound as it contains a cobalt(II) and a cobalt(III) or an average oxidation state of Co^+2.5^. In this case the rate of electron transfer between the two cobalt atoms would be slow as this is a Class I intervalence compound as the metals are nor in identical structural fields. However a small change in crystallization conditions (presence of a two-fold rotation axis) would mean that the compound was a Class III intervalence system with a non-localized electron.

### 3.4. Correlation of Spin State with Structural Parameters

Structural signatures of high spin (HS) and low spin (LS) iron(II) and iron(III) [53] tripodal tren imidazole complexes have been investigated previously from experimental and computational approaches [54,55]. The signatures are 1) the Fe-N_imidazole_ and Fe-N_imine_ bond distances 2) the N_imidazole_-Fe-N_imine_ bite angle 3) the N_imidazole_-Fe-N_imine’_ trans angle 4) the Fe-N_ap_ distance and 5) the N_ap_ conformation. In general the HS state correlates with long Fe-N_imidazole_ and Fe-N_imine_ bond distances (> 2.10 Å), N_imidazole_-Fe-N_imine_ bite angles of ~76°, N_imidazole_-Fe-N_imine’_ trans angles of ~166°, short M-N_ap_ distances (<3.1 Å) and the conformation of the apical nitrogen atom of the tren bent in (“Nin”)towards the iron atom. In contrast the LS state correlates with short Fe-N_imidazole_ and Fe-N_imine_ bond distances (<2.00 Å), N_imidazole_-Fe-N_imine_ bite angles of ~81°, N_imidazole_-Fe-N_imine’_ trans angles of ~175°, long Fe-N_ap_ distances (>3.1 Å) and “N out” or planar conformation of the apical tren nitrogen atom. The above structural signatures are so pronounced that LS structures of iron(II) and iron(III) are essentially identical with these ligands as are HS structures. In other words the effect of oxidation state is minor relative to spin state on structural parameters. 

Comparison of the above structural parameters of iron(III) (d^5^) and iron(II) (d^6^) with the analogous values for all of the cobalt(III) (d^6^) complexes in Table 2 clearly suggests that all of the cobalt(III) complexes are LS. This is hardly surprising as all but a few cobalt(III) complexes are LS. This does not mean that structural data is a true measurement of the electronic ground state but it does suggest that the above parameters correlate extremely well with spin state selection in this class of complexes. The [Cotren(MePyrzH)_3_][Cotren(MePyrz)_3_]I requires further comment as it contains both a cobalt(II) and a cobalt(III). The Co-pyrazole bond distance is the longest (2.048 Å) and the bite (77.2°) and trans (172.1°) angles are the smallest for Cotren(MePyrzH)_3_ of all the complexes listed in Table 2. The lengthening of the Co-pyrazole bond distance can be explained on the fact that pyrazolate is a stronger binder than a pyrazole. The bite and trans angles are not completely in the LS regime but have moved in that direction. More definitive comments on the spin state of the Co(II) in this complex requires a separate investigation to synthesize, isolate and magnetostructurally characterize a mononuclear [Cotren(MePyrzH)_3_]^2+^ species. It is possible that the Co(II) d^7^ species is LS or close to the equilibrium:

^2^E (LS) ⇌ ^4^T (HS) spin crossover point. (3)

## 4. Summary

One aspect of materials science work is to incorporate features into a molecular system that have potential for applications. Two aspects that may be desirable to incorporate into a molecular system are spin crossover (SC) and intervalence (IV). A SC molecule can switch between two electronic states with different magnetic properties due to a change in temperature, pressure, optical stimulation or other environmental features such as pH. Such molecules can be thought of as a switch or memory storage device. A metal(II)-metal(III) intervalence molecule has a non integral average oxidation state and can serve as a storage site for an electron or could be used to promote rapid electron transfer. If the symmetry of the intervalence compound resulted in a single metal in the asymmetric unit then the compound would be a Class III intervalence species in which the electron is not localized and the metal ions are in an average non integral oxidation state. Well know examples of these are magnetite, Fe_3_O_4,_ basic iron acetate, Fe_3_O (OAc)_6_(H_2_O)_3_, and the Creutz Taube ion, [(NH_3_)_5_Ru(pyrazine)Ru(NH_3_)_5_]^5+^. The pyrazole dimers/pseudodimers discussed here have the features of SC and IV. In addition they have the ability to self-assemble through a stereochemical (chiral) molecular recognition process which means that one tren pyrazole complex can bind selectively to only the same enantiomer of a racemic mixture. If these molecules were incorporated into a material then the door is open to fully exploit the stereochemical and symmetry features as well as the SC and IV aspects. An example of how this incorporation could be achieved is as follows. The central nitrogen atom of the tren can be alkylated by reaction with an alkyl iodide, RI, or other agent. A silicon (or other) based material, which had incorporation of this functionalization, -Si-Si-C(I)-Si-Si- could be reacted with tren to give -Si-Si-C(N(CH_2_CH_2_NH_2_)_3_–Si-Si-. Further reaction of this with MepyrzH followed by a metal would incorporate half of the dimer (monomer) into the material The other half of the dimer can self-assemble onto the first half by chiral recognition and formation of hydrogen bonds to give dimer/pseudodimer attached to the original material. Incorporation of the dimer/pseudodimer into the original material allows for possible exchange reactions such as selective removal of a single enantiomer from solution. There are many potential uses of such a material that could exploit the SC and IV properties. The complexes presented here and in earlier work show that subtle variations in reaction and or crystallization conditions have a profound effect on the molecular symmetry by altering the space group that is exhibited. Any of these features could be exploited by incorporating these molecules into a new material.

## Figures and Tables

**Table 1:** a) [Cotren(MepyrzH)_3_](ClO_4_)_3_^.^3.5 H_2_O, b) [Cotren(MepyrzH_0.5_)_3_]_2_(ClO_4_)_3_, c) [Cotren(MepyrzH_0.5_)_3_]_2_(BF_4_)_3_, d) [Cotren(MepyrzH)_2_(Mepyrz)} [Cotren(MepyrzH)(Mepyrz)_2_](BF_4_)_3_^.^3.81 H_2_O, e) {[Cotren(MepyrzH_0.5_)_3_]_2_ (ClO_4_)_3_^.^(C_6_H_6_)_2_ (CH_3_CN)_2_ @123K, f) {[Cotren(MepyrzH_0.5_)_3_]_2_ (ClO_4_)_3_^.^(C_6_H_6_)_2_^.^(CH_3_CN)_2_ @150K, g) [Cotren(MepyrzH)_3_][Cotren(Mepyrz)_3_] I_2._

	a)	b)	c)	d)	e)	f)	g)
Empirical formula	C_21_H_37_Cl_3_Co N_10_O_15.5_	C_42_H_57_Cl_3_Co_2_ N_20_O_12_	C_42_H_57_B_3_Co_2_ F_12_ N_20_	C_42_H_64.62_B_3_Co_2_ F_12_N_20_O_3.81_	C_58_H_75_Cl_3_Co_2_ N_22_O_12_	C_58_H_75_Cl_3_Co_2_ N_22_O_12_	C_42_H_57_Co_2_ N_20_I_2_
M/g mol^−1^	842.89	1258.28	1220.37	1292.43	1496.61	1496.61	1213.74
Temperature/K	295(2)	123(2)	123(2)	150(2)	123(2)	150(2)	150(2)
λ/Å	0.71073	0.71073	1.54178	0.71073	0.71073	0.71073	0.71073
Crystal System	Monoclinic	Trigonal	Trigonal	Monoclinic	Tetragonal	Tetragonal	Monoclinic
Space group	P21//c	R32	R32	Cc	P4_3_2_1_2	P4_3_2_1_2	Cc
Unit cell dimensions	a = 12.9005(9) Å	a = 16.7663(2) Å	a = 16.6003(3) Å	a = 16.268(2) Å	a = 11.82570(10) Å	a = 11.8445(18) Å	a = 21.1716(10) Å
	b = 17.8994(10) Å	b = 16.766(3) Å	b = 16.6003(3) Å	b = 17.394(2) Å	b = 11.82570(10) Å	b = 11.8445(18) Å	b = 12.3416(6) Å
	c = 15.5785(11) Å	c = 20.0641(3) Å	c = 20.0513(6) Å	c = 20.188(3) Å	c = 48.5723(11) Å	c = 48.694(8) Å	c = 19.6850(9) Å
	α = 90°	α = 90°	α = 90°	α = 90°	α = 90°	α = 90°	α = 90°
	β = 99.931(7)°	β = 90°	β =90°	β = 106.941(2)°	β = 90°	β = 90°	β = 93.1640(10) °
	γ = 90°	γ = 120°	γ = 120°	γ = 90°	γ = 90°	γ = 90°	γ = 90°
Volume/Å^3^	3543.3(4)	4884.55(14)	4785.25(19)	5464.7(13)	6792.70(17)	6831.4(18)	5135.7(4))
Z	4	3	3	4	4	4	4
Abs. Coeff./mm^−1^	0.791	0.697	4.776	0.710	0.682	0.679	1.900
F(000)	1740	1950	1878	2664	3112	3112	2436
Crystal size/mm^3^	0.53 × 0.47 × 0.08	0.47 × 0.43 × 0.28	0.26 × 0.24 × 0.19	0.295 × 0.05 × 0.045	0.39 × 0.39 × 0.32	0.21 × 0.30 × 0.44	0.22 × 0.135 × 0.11
Theta range/^o^	5.05 to 26.37	5.164 to 32.828	5.33 to 75.57	1.76 to 22.50	3.05 to 35.17	2.13 to 25.00	1.91 to 27.50
Index ranges	−15 ≤ h ≤ 16	−24 ≤ h ≤ 25	−20 ≤ h ≤ 20	−17 ≤ h ≤ 17	−19 ≤ h ≤ 18	−14 ≤ h ≤ 13	−27 ≤ h ≤ 27
	−22 ≤ k ≤ 19	−25 ≤ k ≤ 25	−19 ≤ k ≤ 20	−18 ≤ k ≤ 18	−18 ≤ k ≤ 18	−14 ≤ k ≤ 14	−16 ≤ k ≤ 16
	−18 ≤ l ≤ 19	−30 ≤l ≤ 30	−14 ≤ l ≤ 25	−21 ≤ l ≤ 21	−76 ≤ l ≤ 51	−57 ≤ l ≤ 57	−25 ≤ l ≤ 25
Reflections Collected	20,906	31,672	11,282	15,317	99,409	57,503	36,049
Independent Reflections	7193	3862	2195	6970	14,527	6006	11,652
R1	0.0853	0.0294	0.0814	0.0775	0.0724	0.0537	0.0371
WR2	0.2031	0.0847	0.2203	0.1913	0.1515	0.1189	0.0774
GOF on F^2^	1.031	1.123	1.075	1.074	1.194	1.000	1005

**Table 2:** Selected bond distances (Å) and angles (°) for a) [Cotren(MepyrzH)_3_](ClO_4_)_3_^.^3.5 H_2_O, b) [Cotren(MepyrzH_0.5_)_3_]_2_(ClO_4_)_3_ [a], c) [Cotren(MepyrzH_0.5_)_3_]_2_(BF_4_)_3,_ [a] d) [Cotren(MepyrzH)_2_(Mepyrz)}[Cotren(MepyrzH)(Mepyrz)_2_ ](BF_4_)_3_^.^3.81 H_2_O [b], e) {[Cotren(MepyrzH_0.5_)_3_]_2_ (ClO_4_)_3_^.^(C_6_ H_6_)_2_^.^(CH_3_CN)_2_ @123K [c], f) {[Cotren(MepyrzH_0.5_)_3_]_2_ (ClO_4_)_3_^.^(C_6_H_6_)_2_^.^(CH_3_CN)_2_ @150K, [c] g) [Cotren(MepyrzH)_3_][Cotren(Mepyrz)_3_] I_2_ [d].

	a)	b)	c)	d) [CoH_2_L]^2+^ and [CoHL]^+^	e)	f)	g) [CoH_3_L]^2+^ and [CoL]
SG and sym	P2_1/c_ C_2h_	R32 D_3_	R32 D_3_	Cc, Cs	P4_3_2_1_2 D_4_	P4_3_2_1_2 D_4_	Cc, Cs
T(K)	295(2)	123(2)	123(2)	150(2)	123(2)	150(2)	150(2)
Co-N_ap_ non-bonded	3.339	3.468	3.468	3.432 3.462	3.492	3.492	3.306 3.300
M-M’non-bonded	NA	5.853	5.853	5.773	5.861	5.872	5.847
Co-N1(imine)	1.954(2)	1.9591(14)	1.962(4)	1.954(18) 1.947(18)	1.951(2)	1.963(4)	2.031(8) 2.060(7)
	1.955(2)			1.940(17) 1.982(17)	1.961(2)	1.951(4)	2.049(7) 2.067(8)
	1.970(3)			1.974(18) 1.921(18)	1.924(2)	1.962(4)	2.064(9) 2.038(10)
average	1.960	1.9591	1.9624	1.956 1.950	1.945	1.959	2.048 2.055
Co-N2(pyrazole)	1.908(3)	1.9141(15)	1.910(4)	1.896(15) 1.918(15)	1.908(2)	1.928(4)	1.979(7) 2.017(8)
	1.919(2)			1.893(16) 1.8959(16)	1.917(2)	1.913(4)	2.019(8) 2.048(7)
	1.928(2)			1.954(17) 1.883(17)	1.924(2)	1.915(4)	2.145(5) 1.865(5)
average	1.918	1.9141	1.910	1.914 1.899	1.916	1.919	2.048 1.977
N1-Co-N2 (bite)	81.32(10)	81.66(6)	81.52(17)	81.6(8) 83.6(8)	82.16(10)	80.94(15)	77.3(3) 80.0(3)
	81.53(11)			82.5(7) 81.3(7)	81.32(10)	82.00(15)	78.3(3) 80.6(3)
	81.46(11)			80.2(8) 80.1(7)	81.25(9)	81.17(16)	76.1(3) 82.2(3)
average	81.44	81.66	81.52	81.4 81.7	81.58	81.37	77.2 80.9
N1-Co-N2’(trans)	175.82(11)	175.23(6)	175.43(17)	175.0(7) 176.0(8)	174.95(11)	173.78(17)	174.2(4) 175.8(3)
	175.22(11)			174.0(8) 177.5(8)	174.19(10)	174.71(17)	170.6(3) 176.2(3)
	175.19(11)			172.6(8) 173.7(8)	175.16(10)	175.12(16)	171.4(3) 172.9(3)
average	175.4	175.23	175.43	173.9 175.7	174.77	174.54	172.1 174.97
C1-N_ap_-C1	118.6(3)	119.991(5)	119.996(14)	121(2) 130(2)	120.0(2)	120.3(4)	119.6(80 116.2(8)
	120.8(3)	119.990(6)	119.991(12)	114(2) 116.0(19)	120.6(2)	120.0(4)	121.1(8) 120.7(7)
	119.1(3)	119.984(5)	119.995(14)	123(2) 114(2)	119.4(3)	119.7(4)	116.6(8) 119.1(9)
average	119.5	119.988	119.994	119.3 120.0	120.0	120.0	119.1 118.7

[a] There is only one Co and one arm of tren in the asymmetric unit. [b] There are two Co atoms each with three tren arms in the asymmetric unit, [CoH_2_L]^2+^ (listed first) and [CoHL]^+^ listed second. [c] There is one Co atom and three tren arms in the asymmetric unit. [d] There are two Co atoms each with three tren arms in the asymmetric unit, [CoH_3_L]^2+^ (listed first) and [CoL] listed second.

**Table 3:** Pyrazole hydrogen bond distances (Å) and angles (°) for, [Cotren(MepyrzH_0.5_]_2_ (ClO_4_)_3_, [Cotren(MepyrzH_0.5_]_2_ (BF_4_)_3,_ [Cotren(MepyrzH)_2_(Mepyrz)][Cotren(MepyrzH)(Mepyrz)_2_](BF_4_)_3_^.^3.81 H_2_O, {[Cotren(MepyrzH_0.5_)_3_]_2_}(ClO_4_)_3_^.^(C_6_H_6_)_2_^.^(CH_3_CN)_2_ @123K, {[Cotren(MepyrzH_0.5_)_3_]_2_} (ClO_4_)_3_^.^(C_6_H_6_)_2_^.^(CH_3_CN)_2_ @150K, [Cotren(MepyrzH)_3_][Cotren(Mepyrz)_3_] I_2._

Compound	Interaction	d(D-H)	d(H ^…^A)	d(D ^…^A)	<(DHA)
[Cotren(MepyrzH_0.5_]_2_ (ClO_4_)_3_	N-H^….^N’ (three)	0.88	1.83	2.670(3)	160.1
T = 123(2)K					
Average		0.88	1.83	2.670	160.1
[Cotren(MepyrzH_0.5_]_2_ (BF_4_)_3_	N-H^….^N’ (three)	0.88	1.83	2.674(8)	158.9
T = 123(2)K					
Average		0.88	1.83	2.67	158.9
[Cotren(MepyrzH)_2_(Mepyrz)}[Cotren(MepyrzH)(Mepyrz)_2_](BF_4_)_3_^.^3.81 H_2_O	N-H^….^N’ N-H ^…^N’ N-H ^…^N’	0.88	1.88	2.73(2)	162
		0.88	1.87	2.72(3)	161
T = 150(2) K		0.88	1.87	2.714(10)	160.
			
Average		0.88	1.87	2.72	161
{[Cotren(MepyrzH_0.5_)_3_]_2_(ClO_4_)_3_^.^(C_6_H_6_)_2_^.^(CH_3_CN)_2_@123K	N-H^….^N’ N-H ^…^N’ N-H^….^N’	0.88	1.83	2.679(3)	161.9
		0.88	1.83	2.679(3)	161.5
T = 123(2) K		0.88	1.80	2.643(4)	160.5
			
Average		0.88	1.82	2.667	161.3
{[Cotren(MepyrzH_0.5_)_3_]_2_ (ClO_4_)_3_^.^(C_6_ H_6_)_2_^.^ (CH_3_CN)_2_ @150K,	N-H^….^N’ N-H ^…^N’(twice)	0.88	1.81	2.655(7)	160.0
		0.88	1.84	2.685(5)	161.0
T = 150(2) K					
			
Average		0.88	1.83	2.675	160.7
[Cotren(MepyrzH)_3_][Cotren(Mepyrz)_3_] I_2_	N-H^….^N’ N-H ^…^N’ N-H ^….^N’	0.88	1.98	2.819(12)	160.
		0.88	1.89	2.744(12)	162
T = 150(2) K		0.88	1.90	2.754(8)	162
			
Average		0.88	1.92	2.772	152
